# Simulation of Novel Nano Low-Dimensional FETs at the Scaling Limit

**DOI:** 10.3390/nano14171375

**Published:** 2024-08-23

**Authors:** Pengwen Guo, Yuxue Zhou, Haolin Yang, Jiong Pan, Jiaju Yin, Bingchen Zhao, Shangjian Liu, Jiali Peng, Xinyuan Jia, Mengmeng Jia, Yi Yang, Tianling Ren

**Affiliations:** 1 School of Integrated Circuits, Tsinghua University, Beijing 100084, China; guopengwen@mail.tsinghua.edu.cn (P.G.); panj23@mails.tsinghua.edu.cn (J.P.); yjj22@mails.tsinghua.edu.cn (J.Y.); zhaobc22@mails.tsinghua.edu.cn (B.Z.); liushang22@mails.tsinghua.edu.cn (S.L.); pengjl@tsinghua.edu.cn (J.P.); 2 Beijing National Research Center for Information Science and Technology (BNRist), Tsinghua University, Beijing 100084, China; 3 School of Materials Science and Engineering, University of Science and Technology Beijing, Beijing 100083, China; u202140623@xs.ustb.edu.cn; 4 Department of Chemistry, Tsinghua University, Beijing 100084, China; yanghl22@mails.tsinghua.edu.cn; 5 Xingjian College, Tsinghua University, Beijing 100084, China; jia-xy21@mails.tsinghua.edu.cn; 6 Beijing Key Laboratory of Micro-Nano Energy and Sensor, Center for High-Entropy Energy and Systems, Beijing Institute of Nanoenergy and Nanosystems, Chinese Academy of Sciences, Beijing 101400, China; jiamengmeng@ucas.ac.cn; 7 School of Nanoscience and Technology, University of Chinese Academy of Sciences, Beijing 100049, China

**Keywords:** 2D materials, CNT, sub-3 nm node, nanosheet FET, FinFET, GAAFET, TCAD simulation

## Abstract

The scaling of bulk Si-based transistors has reached its limits, while novel architectures such as FinFETs and GAAFETs face challenges in sub-10 nm nodes due to complex fabrication processes and severe drain-induced barrier lowering (DIBL) effects. An effective strategy to avoid short-channel effects (SCEs) is the integration of low-dimensional materials into novel device architectures, leveraging the coupling between multiple gates to achieve efficient electrostatic control of the channel. We employed TCAD simulations to model multi-gate FETs based on various dimensional systems and comprehensively investigated electric fields, potentials, current densities, and electron densities within the devices. Through continuous parameter scaling and extracting the sub-threshold swing (SS) and DIBL from the electrical outputs, we offered optimal MoS_2_ layer numbers and single-walled carbon nanotube (SWCNT) diameters, as well as designed structures for multi-gate FETs based on monolayer MoS_2_, identifying dual-gate transistors as suitable for high-speed switching applications. Comparing the switching performance of two device types at the same node revealed CNT’s advantages as a channel material in mitigating SCEs at sub-3 nm nodes. We validated the performance enhancement of 2D materials in the novel device architecture and reduced the complexity of the related experimental processes. Consequently, our research provides crucial insights for designing next-generation high-performance transistors based on low-dimensional materials at the scaling limit.

## 1. Introduction

**Figure 1:**
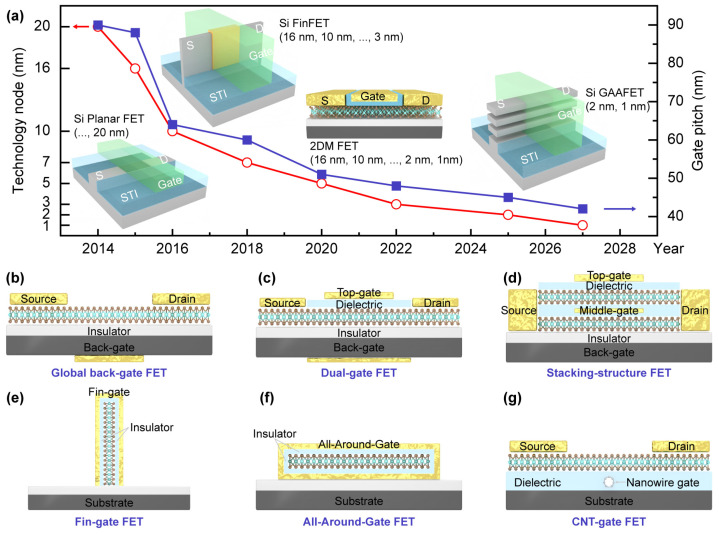
(**a**) Scaling trends of transistor dimensions and gate pitch. STI is shallow trench isolation, which is used to isolate neighboring devices. S and D represent the source and drain of the transistor, respectively. Introduction of new architecture and new materials, (**b**) global back-gate MoS_2_ (CNT) FET, (**c**) local top-gate MoS_2_ FET, (**d**) two-level stacking nanosheet MoS_2_ GAAFET, (**e**,**f**) MoS_2_-based Fin-FET and GAA-FET, (**g**) MoS_2_ FET with buried CNT, respectively.

As the semiconductor industry continues to push the boundaries of Moore’s Law [1], the scaling of FETs to sub-10 nm nodes presents unprecedented challenges [2,3,4]. Traditional silicon-based metal-oxide-semiconductor field-effect transistors (MOSFETs) face severe limitations as device dimensions approach atomic scales. The most critical issue is the intensification of short-channel effects (SCEs) [5], where the electrostatic control of the channel by the gate electrode is compromised, leading to a host of detrimental effects. Drain-induced barrier lowering (DIBL) [6] becomes more severe, causing the threshold voltage to decrease with the reducing channel length and increasing drain voltage. This results in higher off-state currents and degraded subthreshold swing, ultimately limiting the device’s ability to switch off effectively. In addition, quantum mechanical tunneling between the source and drain becomes significant, further exacerbating leakage currents. To address these scaling limitations, the semiconductor industry has explored various innovative device architectures and materials, as shown in Figure 1a. The transition from planar MOSFETs to multi-gate structures such as FinFETs marked a significant milestone in maintaining electrostatic integrity at smaller nodes. However, as the nodes approach the sub-3 nm regime, even FinFETs struggle to provide adequate channel control [7,8].

Low-dimensional materials and novel device architectures have emerged as promising candidates to extend Moore’s Law beyond the limitations of silicon. Two-dimensional (2D) materials, such as transition metal dichalcogenides (TMDs) and black phosphorus, offer unique advantages for ultra-scaled devices [9,10,11,12]. Their atomically thin nature provides excellent electrostatic control, mitigating SCEs more effectively than bulk semiconductors. Moreover, the absence of dangling bonds at the surface of 2D materials reduces interface scattering and trap states, potentially leading to higher carrier mobility and improved subthreshold characteristics [13]. Carbon nanotubes (CNTs) represent another class of low-dimensional materials with exceptional potential for scaled transistors [14,15,16,17,18]. Their one-dimensional structure enables the near-ballistic transport of carriers and superior electrostatic control. Recent advancements in CNT purification and alignment techniques have paved the way for high-performance CNT-based FETs that can compete with silicon devices at advanced nodes. In terms of device architecture, gate-all-around FETs (GAAFETs) [19] and nanosheet FETs have gained significant attention for sub-3 nm nodes [20]. These structures provide enhanced gate control by surrounding the channel material on all sides, effectively suppressing SCEs and enabling further scaling. The ability to stack multiple nanosheets vertically also allows for increased current drive per unit area, addressing the challenges of power density and performance at ultra-scaled nodes. The integration of low-dimensional materials with advanced device architectures opens up new possibilities for overcoming the scaling limitations of traditional Si-based technologies [21,22]. For instance, 2D material-based GAAFETs could potentially combine the benefits of atomically thin channels with superior electrostatic control, resulting in devices with excellent short-channel characteristics and high carrier mobility.

In addition, to fully explore the potential of these novel materials and architectures, technology computer-aided design (TCAD) simulations play a crucial role. Advanced TCAD tools enable the modeling of quantum effects, carrier transport mechanisms, and electrostatics in nanoscale structures with unprecedented accuracy. By simulating various device configurations and material combinations, researchers can optimize designs and predict performance at the scaling limit, guiding the development of next-generation semiconductor devices.

In this study, we employed advanced TCAD simulation techniques to investigate the electrical performance and SCEs of FETs based on 1D/2D materials, including CNT FETs, nanosheet FETs, and GAAFETs (Figure 1b–g) at the scaling limit. By modeling the thinning of the channel layer and scaling of the gate length, we quantified the dependency of 2D MoS_2_ layer thickness and SWCNT diameter on the node, providing valuable guidance for subsequent experimental process design. Comparisons of the switching performance of various multi-gate FETs based on monolayer MoS_2_ at sub-10 nm nodes identified the dual-gate transistor as having an advantage as a high-frequency switching device due to its larger effective gate length. Visualization of the behavior of FinFETs and GAAFETs in the on-state, along with comparisons of the simulated performance metrics of novel devices and traditional silicon FinFETs, validated the promise for overcoming the scaling limitations of traditional CMOS technology, enabling further miniaturization and performance enhancement of future semiconductor devices.

## 2. Simulation Method

The 2022 version of Sentaurus TCAD software was utilized in this research, which is a widely used tool in semiconductor device simulation. The software incorporates various physical models for semiconductor processes, enabling the simulation of electrical characteristics and facilitating computer-aided design. The latest version supports atomic-scale modeling and integrates with QuantumATK, allowing for the simulation of novel materials and advanced device structures. It also enhances electrical characterization by incorporating quantum effects, ballistic transport, and stress engineering models, supporting the optimization of advanced logic, memory, and power devices. To achieve realistic results, all the simulations are conducted by including the Slotboom model, Shockley Read Hall model, as well as mobility models of High Field Saturation and Enormal. Specific simulation parameters are listed in detail in the discussion section.

## 3. Results

### Formatting of Mathematical Components

(1) $$\mathrm{SS}=\frac{\partial V_{\mathrm{GS}}}{\partial \mathrm{lg}I_{\mathrm{DS}}}$$

where *V*_GS_ and *I*_DS_ represent gate-source voltages and drain-source current, respectively.

(2) $$\mathrm{DIBL}=\frac{V_{\mathrm{th}}^{high}-V_{\mathrm{th}}^{low}}{V_{\mathrm{DS}}^{high}-V_{\mathrm{DS}}^{low}}$$

where *V*_DS_ and *V*_th_ represent source-drain voltages and the corresponding threshold voltage, respectively.

(3) $$E_{g}=0.85/d_{\mathrm{SWCNT}}$$

where *E*_g_ and *d*_SWCNT_ represent bandgap and diameter of SWCNT.

## 4. Discussion

**Figure 2:**
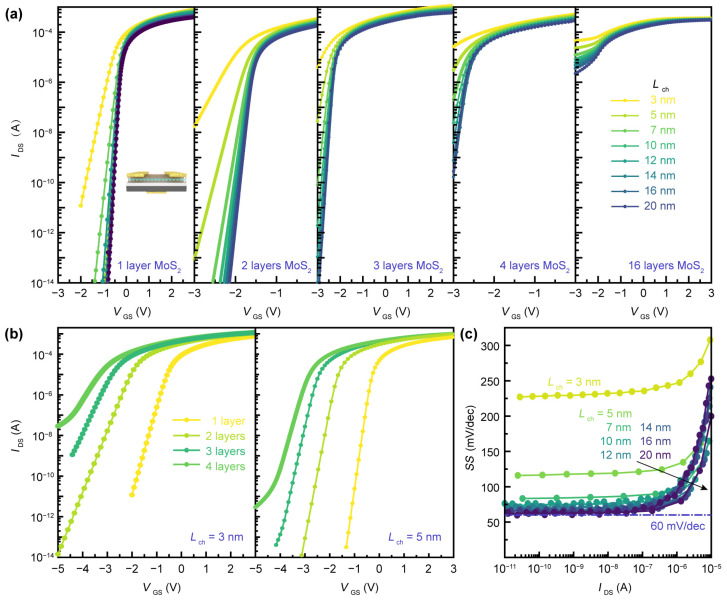
(**a**) The transfer curves of global back-gate MoS_2_-based FET with different number of layers. HfO_2_ is used for the gate insulator with a thickness of *T*_ox_ = 3 nm. The length of source (drain) is equivalent to channel length *L*_ch_. Channel width *W*ch = 8 nm, and drain-source voltage *V*_DS_ = 100 mV. (**b**) Layer-dependent characteristics of transfer curves of global back-gate MoS_2_ FET with 3 nm and 5 nm channel length, respectively. (**c**) The extracted sub-threshold swing SS from (**a**) as a function of drain current *I*_DS_. SS limitations are above 60 mV dec^−1^ at room temperature.

The simulation results of the back-gated MoS_2_ FET with doped silicon as the substrate and gate are shown in Figure 2. MoS_2_ predominantly exhibits intrinsic n-type doping due to interface charge impurities and sulfur vacancy defects, and the Fermi level can be affected by n-type doping of MoS_2_ (10^20^ cm^−2^ in this stimulation, and the related doping parameters can be seen in Table 1), which is manifested as an upward shift in the Fermi level and a change in the band structure, inducing significant affects in the electronic and optical properties of MoS_2_. Therefore, we maintained a fixed doping concentration and the typical n-type doping characteristics in this simulation to avoid other effects. HfO_2_ emerges as an ideal dielectric material due to its low equivalent oxide thickness (EOT) of 0.51 nm (modeling parameters refer to [23]). Research analyzing HfO_2_ defect energy levels has demonstrated its good compatibility with MoS_2_, reducing the impact of defect levels on carrier transport. 

As illustrated in Figure 2a, as the channel length decreases from 20 nm to 3 nm, the switching characteristics of FETs with different channel layer numbers deteriorate, while the saturation current remains relatively unchanged. The impact of channel length becomes more pronounced with increasing layer numbers, with single-layer MoS_2_ channels exhibiting better resistance at this point. Conversely, as the number of layers increases, gate control capability significantly weakens. From single-layer to four-layer MoS_2_, FETs increasingly tend towards depletion mode, with 16-layer MoS_2_ channel FETs unable to be effectively turned off at a gate voltage of −3 V.

To thoroughly investigate the application potential of MoS_2_ in short-channel devices, we compared the transfer curves of back-gated FETs with channel lengths of 3 nm and 5 nm, using MoS_2_ channels ranging from single-layer to four-layer, as shown in Figure 2b. Compared to the 3 nm channel, the 5 nm channel length exhibits reduced SCEs, resulting in noticeably optimized device switching characteristics. Furthermore, the transfer characteristic curves of both the 3 nm and 5 nm channel devices reveal that the impact of MoS_2_ layer number on device performance increases non-linearly as the number of layers decreases.

For MOSFETs, the inherent limitations of carrier thermal radiation result in a subthreshold swing (SS) that remains above 60 mV/dec at room temperature, which can be expressed as Equation (1). The SS of FETs with single-layer MoS_2_ channels is shown in Figure 2c. For channel lengths above 7 nm, devices exhibit SS close to the Boltzmann theoretical limit (60 mV/dec), which is comparable to the experimental results [24,25]. However, the 3 nm and 5 nm channel devices show larger SS due to increasingly significant SCEs, with the SS of 3 nm channel devices exceeding 200 mV/dec, indicating substantial room for improvement. Identifying dielectric materials with smaller EOT can effectively enhance device switching characteristics. Additionally, in short-channel devices, semiconductor carriers exhibit near-ballistic transport, dissipating almost all their energy upon contact. Therefore, improving the contact between two-dimensional materials and metals is particularly crucial. Consequently, the selection of contact metals is one of the effective measures to improve device switching characteristics.

**Figure 3:**
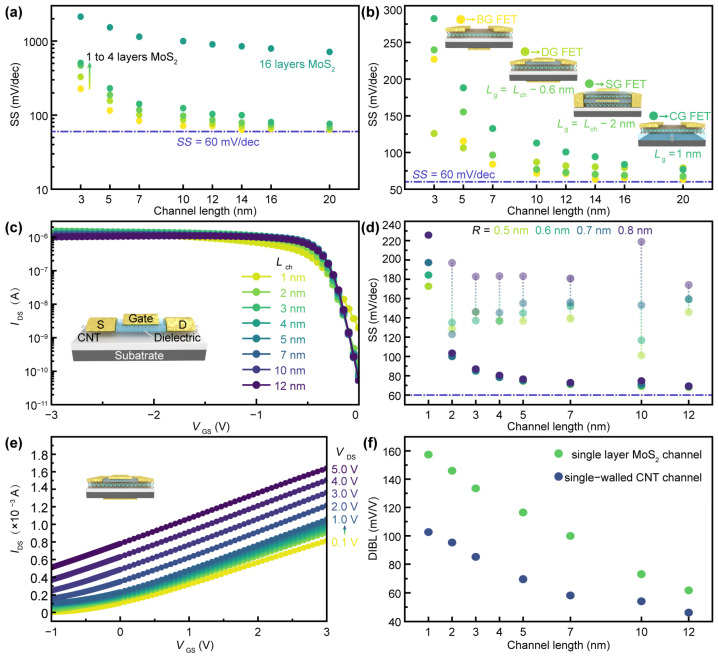
(**a**) Layer-dependent characteristics of SS for MoS_2_-based back-gate FET with various channel length. (**b**) SS as a function of channel length for four types of planar-gate FETs. BG, DG, SG, and CG represent back-gate, dual-gate, stacking-gate, and CNT-gate for MoS_2_-based FET, respectively. (**c**) SWCNT as channel materials in global back-gate FET and channel length dependent transfer curves. HfO_2_ is used for the gate insulator with a thickness of *T*_ox_ = 1.8 nm. The width of source (drain) *W*_SD_ = 2 nm and the radius of CNT *R* = 0.5 nm. (**d**) The relationships between SS and channel length extracted from (**c**) for CNT-based FET with various radii. (**e**) The linear transfer curves of CNT FET with a diameter of 1 nm at varying *V*_DS_. (**f**) DIBL comparison between single layer MoS_2_ and single-walled CNT as the channel of back-gate FETs in varying channel length, which indicates the short-channel effects within sub-10 nm.

As the channel length reaches its physical limit, the thickness of the semiconductor is also required to be scaled to the atomic scale to minimize SCEs [26]. The SS at a channel length of 3 nm is extracted from the transfer curve shown in Figure 2a, as illustrated in Figure 3a. As the thickness of the MoS_2_ channel decreases from 10.4 nm (16 layers) to 0.65 nm (single layer), the SS of the FET approaches the SS limit as the channel lengths exceed 10 nm. This performance improvement is attributed to the high carrier mobility of 2D materials, which remains unaffected by the reduction in thickness. However, the performance of the 16-layer device is not ideal. Therefore, in subsequent simulations, unless otherwise specified, single-layer MoS_2_ is used as the selected 2D material. Figure 3b illustrates the dependence of SS on channel length for four types of planar-gate FETs. In the case of dual-gate FETs, the top-gate dielectric and electrode are slightly narrower than the channel length, corresponding to a gate length (i.e., technology node) of *L*_g_ = *L*_ch_ − 0.6 nm. For the stacking-gate FETs, a middle-gate is employed to apply a variable electrostatic field for gate control; due to the surrounding gate dielectric, its effective gate length is 2 nm shorter than the channel length. For FETs with a buried single-walled carbon nanotube (SWCNT) of 1 nm in diameter, the gate length is fixed at 1 nm. When the channel length exceeds 10 nm, all four types of gate-controlled FETs exhibit good SS characteristics. However, as the channel length approaches 3 nm, the gate lengths of the three FETs with local gates (dual-gate, stacking-gate, CNT-gate) approach their scaling limits, measuring 2.4 nm, 1 nm, and 1 nm, respectively. The dual-gate FET has the minimum SS of 126 mV/dec, which corresponds to a larger on/off rate than the other gate-controlled FETs, likely due to the efficient gate control provided by the longer gate length.

In addition to serving as a record-breaking short gate, CNTs can also be used as channel materials due to their excellent electrical properties, such as high carrier mobility and room-temperature ballistic transport [15,17,18]. In this study, due to the functional limitations of the simulation software, it is challenging to fully incorporate the specific morphology of CNTs in the simulation. Given that CNTs are utilized as the channel material in our device simulations, we have assumed that all the carbon nanotubes employed are of the semiconductor type. To approximate the actual characteristics of the CNTs as closely as possible, we have calculated the band gaps for carbon nanotubes with different diameters based on established formulas (Equation (3)) [27].

The intrinsic CNT FETs show p-type characteristics, as depicted in Figure 3c. For a top-gate FET with a 1 nm diameter SWCNT, the on-current approaches the microampere level, which is consistent with experimental results. Furthermore, for CNT FETs with gradually increasing diameters, the SS is extracted for channel lengths ranging from 1 nm to 12 nm (corresponding to Lg from 0.4 nm to 11.4 nm), as shown in Figure 3d. Even when the gate length is reduced to 2.4 nm, the SS remains very low (85 mV/dec), attributed to the effective channel gate control and suppressed direct tunneling between the source and the drain, indicating the significant advantages of CNTs as a single-channel material candidate of Si-based integrated circuits. Additionally, when the channel length exceeds 2 nm (with a gate length of 1.4 nm), the impact of SWCNT diameter on SS becomes negligible, making it more compatible with processing techniques for materials of varying dimensions for required high switching speed electronics.

As a manifestation of the SCEs in MOSFETs, DIBL is observed when the gate length is short; the slope of the current versus gate voltage curve decreases, and the gate may even fail to turn off the device completely [28]. The formula of DIBL can be expressed as Equation (2). Therefore, to quantitatively assess DIBL, the threshold voltage of the FET must be obtained under both high and low source–drain voltages. Figure 3e shows the transfer characteristics of a MoS_2_ dual-gate FET represented in linear form, with the lowest and highest source–drain voltages being 0.1 V and 5 V, respectively. Simulations have been used to study the impact of channel length (gate length) scaling on DIBL (Figure 3f). As the gate length decreases, both single-layer MoS_2_ and SWCNT-based top-gate FETs exhibit significant SCEs but still outperform Si-based FinFETs (with a DIBL of approximately 225 mV/V as *L*_g_ = 12 nm). This comparison further indicates the potential of low-dimensional materials in electronic devices. Additionally, the CNT FET demonstrates a lower DIBL effect, likely due to its room-temperature ballistic transport properties.

**Figure 4:**
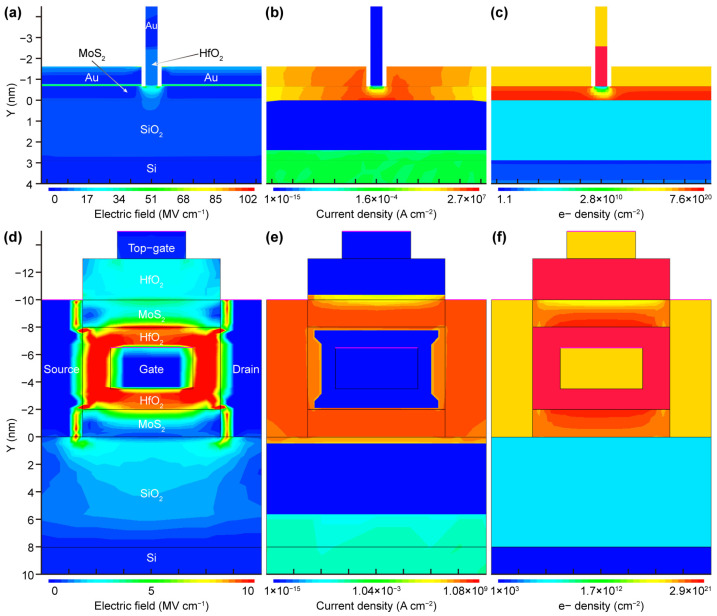
Electric field (**a**), current density (**b**), and electron density (**c**) contour plots in the on-state (*V*_DS_ = 100 mV, *V*_BG_ = 3 V, *V*_TG_ = 3 V) for dual-gate MoS_2_ FET. Electric field (**d**), current density (**e**), and electron density (**f**) contour plots in the on-state (*V*_DS_ = 100 mV, *V*_BG_ = 3 V, *V*_MG_ = 3 V, *V*_TG_ = 3 V) for two-level stacking-gate nanosheet MoS_2_ GAAFET. HfO_2_ is used for the tog-gate and middle-gate insulator with thickness of *T*_ox_ = 1.8 nm and *T*_oxM_ = 1 nm, respectively. The electrical characteristics of both devices were simulated by 2D planar models.

Multi-gate FETs can effectively avoid the capacitance overlap between the source/drain electrodes and the gate. We performed detailed simulations of dual-gate FETs and stacking-gate FETs to understand the electrostatic gate control effect on the devices under applied variable gate voltages. For two-dimensional material electronic devices containing multiple stacked layers and multiple gates, introducing tunneling effects complicates the key issues, so our study does not consider the electric field distribution in the off-state. When the device is in the on-state, due to the low global back-gate voltage applied, the electric field impact on the channel layer is small, so the local top-gate effect is more pronounced. The electric field lines are mainly confined within the MoS_2_, but some field lines penetrate into the underlying dielectric layer (Figure 4a). This phenomenon is more evident in Figure 4d, where under the effective gate control of the middle-gate, high electric fields are distributed in the surrounding dielectric layers. Furthermore, the electric field lines in the underlying MoS_2_ of the stacked layers exhibit a downward radiating decay pattern, penetrating into the SiO_2_ dielectric layer up to a depth of 7 nm, further demonstrating the strong gate control of the middle-gate. Notably, in the MoS_2_ channel above the stacked layers, due to the simultaneous effect of the top-gate and middle-gate, the electric field lines exhibit a symmetric distribution of initial decay followed by enhancement, indicating that the gate control effects of the local top-gate and middle-gate are comparable. The electric field distribution results show that in electronic devices under multiple gate controls, in addition to considering the back-gate screening effect, the necessity of multi-gate coupling must also be considered. Figure 4b,e show the total current density (including electron and hole current density) of the two devices, respectively. As the FET under a fixed source–drain bias (0.1 V), the current density distribution is mainly confined around the variable gate-biased regions, and a conductive “channel” is formed between the source and drain contact points in the MoS_2_ layer, which is the origin of the channel [29]. It should be noted that in the dual-gate FET, the current density increases under the drain contact tip, leading to a slightly different distribution state around the source. The main reason this phenomenon is not obvious in the stacking-gate FET is that the vertical current density change induced by the middle-gate is greater than the horizontal difference. For a single-layer MoS_2_ with a uniform depth channel, the horizontal and vertical current density variations with distance are significantly different, mainly due to the different transport characteristics in the structure. Horizontal transport depends on horizontal mobility, while vertical transport depends on the tunneling of the metal–semiconductor contact, so the drift–diffusion transport model cannot be simply used to explain it. Furthermore, the electron density distribution throughout the entire device was also characterized (Figure 4c,f). The electron density in the entire MoS_2_ channel is relatively high in the on-state [11,16]. The electron density in the dual-gate FET exhibits a radiating distribution under the top-gate, covering the thickness direction, and the horizontal electron density distribution in the channel is wider than the gate length, indicating a larger effective gate length. In contrast, the effective gate length of the stacking-gate FET is smaller, which can also explain the origin of the low SS characteristics in the scaling limit of the dual-gate FET (Figure 3b). The electron density distribution in the thickness direction of the second layer MoS_2_ channel in the stacking-gate FET is not symmetric, although the electric field effect on the second layer by the top-gate and middle-gate is equivalent. Due to the difference in dielectric layers, charge carriers (mainly electrons) form different inversion layers.

**Figure 5:**
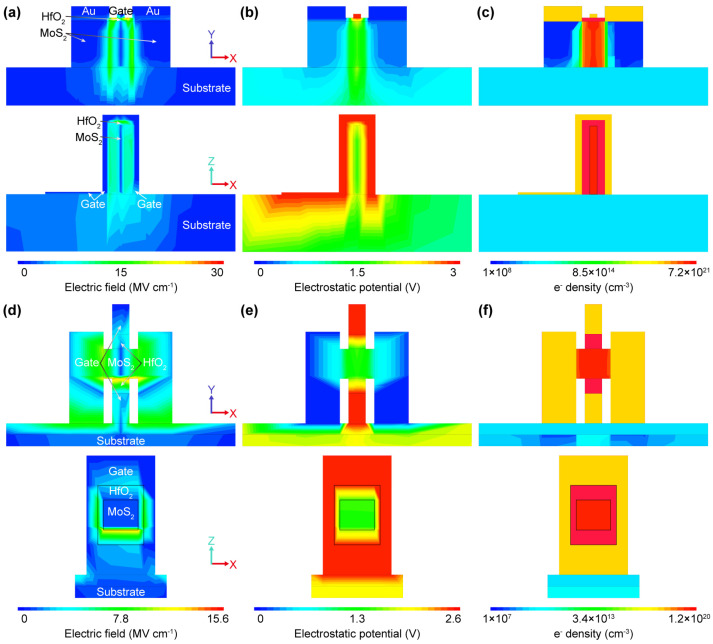
Electric field (**a**), electrostatic potential (**b**), and electron density (**c**) contour plots in the on-state (*V*_DS_ = 100 mV, *V*_GS_ = 3 V) for MoS_2_ FinFET. Electric field (**d**), electrostatic potential (**e**), and electron density (**f**) contour plots in top and cross view in the on-state (*V*_DS_ = 100 mV, *V*_GS_ = 3 V) for MoS_2_ GAAFET. The electrical characteristics of both devices were simulated by 3D models, and the profiles upper and lower each figure are from the XY and XZ directions of the model respectively.

We have established a three-dimensional model to simulate two novel architecture devices—MoS_2_-based FinFETs and GAAFETs (modeling parameters refer to [30,31]). As a small difference in gate parameters of the three-dimensional structure can induce a large change in the electrical characteristics, discussing performance in terms of current alone is meaningless, while electric field distribution can more intuitively demonstrate the efficient gate control of the three-dimensional gate. Figure 5a shows that under the electrostatic field effect of the fin-gate between the source and drain, the MoS_2_ channel layer exhibits an overall symmetric electric field distribution in the horizontal direction, and the electric field strength under the drain contact tip is slightly higher due to the source–drain bias. The electric field near the drain is not very high compared to other channel regions thus greatly reducing hot carrier effects and demonstrating the great potential of novel two-dimensional material devices in reducing SCEs. Under the same gate bias conditions, the electric field in the MoS_2_ GAAFET channel region is lower than that of the FinFET (Figure 5d), indicating that GAAFET has weaker gate control and yet more advantages over FinFET in reducing SCEs, which also applies to Si-based electronics. Figure 5b,e characterize the electrostatic potential distribution in the on-state of the two devices at a source–drain bias of 0.1 V and gate voltage of 3 V. It is worthwhile to note that the actual potential in the channel region is about 1.5 V, indicating that the wide bandgap of MoS_2_ (about 1.8 eV) can withstand higher breakdown voltages compared to narrow bandgap Si-based (about 1.1 eV) devices. It should be noted that since the source–drain bias is much smaller than the gate voltage, there is no obvious potential difference between the drain and source. The electron density contours show that the electron density in the channel material is relatively high [11,16] and, combined with the electric field strength distribution, both indicate that the fin-gate and all-around-gate greatly enhance the carrier concentration in the channel region, leading to a strong inversion in the MoS_2_ channel.

## 5. Conclusions

In summary, we conducted extensive and comprehensive TCAD simulations on multi-gate FETs based on 2D MoS_2_ and 1D CNTs to address the scaling challenges below the 10 nm node. The simulations reveal that as the channel length of 2D MoS_2_ decreases, the switching characteristics of the device significantly deteriorate, but this effect can be mitigated using monolayer MoS_2_. Among multi-gate FETs based on monolayer MoS_2_, dual-gate FETs exhibit higher switching rates due to their larger effective gate length. At the scaling limit, GAAFETs using nanosheets show relatively weaker gate control over the channel compared to FinFETs but offer greater flexibility in process design. FETs using 1D CNTs as the channel material achieve an SS of 95 mV/dec and a DIBL of 100 mV/V at the 1.4 nm node, outperforming devices based on monolayer MoS_2_ and far exceeding the performance of Si-based devices. Additionally, for nodes above 2 nm, the switching performance of CNT FETs is not sensitive to the diameter of SWCNTs, significantly reducing the complexity of experimental design. Our sustainable scaling simulations of 1D/2D multi-gate FETs offer valuable insights for the design and experimentation of next-generation electronic devices.

## Figures and Tables

**Table 1:** Doping parameters of seven types of FETs.

FETs	Doping Density	Channel Length (nm)	Channel Width (nm)	Channel Height (nm)	Number of Dopants (nm^−1^)
BG FET	10^20^ cm^−2^	3	7	0.65	6.50 × 10^5^
DG FET	10^20^ cm^−2^	3	7	0.65	6.50 × 10^5^
SG FET	10^20^ cm^−2^	3	7	0.65	6.50 × 10^5^
SWCNT FET	10^20^ cm^−2^	3	1	1	1.00 × 10^6^
CG FET	10^17^ cm^−3^	3	5	0.65	3.25 × 10^5^
FinFET	10^17^ cm^−3^	3	0.65	6	3.90 × 10^5^
GAAFET	10^17^ cm^−3^	3	5	0.65	3.25 × 10^5^

BG FET, DG FET, SG FET, and CG FET represent back-gate FET, dual-gate FET, stacking-gate FET, and SWCNT-gate FET, respectively.

## Data Availability

Data are contained within the article.
